# Estimated Prevalence of and Factors Associated With Clinically Significant Anxiety and Depression Among US Adults During the First Year of the COVID-19 Pandemic

**DOI:** 10.1001/jamanetworkopen.2022.17223

**Published:** 2022-06-15

**Authors:** Ronald C. Kessler, Christopher J. Ruhm, Victor Puac-Polanco, Irving H. Hwang, Sue Lee, Maria V. Petukhova, Nancy A. Sampson, Hannah N. Ziobrowski, Alan M. Zaslavsky, Jose R. Zubizarreta

**Affiliations:** 1 Department of Health Care Policy, Harvard Medical School, Boston, Massachusetts; 2 Frank Batten School of Leadership & Public Policy, University of Virginia, Charlottesville; 3 Department of Statistics, Harvard University, Cambridge, Massachusetts; 4 Department of Biostatistics, Harvard Chan School of Public Health, Boston, Massachusetts

## Abstract

**Importance:**

Claims of dramatic increases in clinically significant anxiety and depression early in the COVID-19 pandemic came from online surveys with extremely low or unreported response rates.

**Objective:**

To examine trend data in a calibrated screening for clinically significant anxiety and depression among adults in the only US government benchmark probability trend survey not disrupted by the COVID-19 pandemic.

**Design, Setting, and Participants:**

This survey study used the US Centers for Disease Control and Prevention Behavioral Risk Factor Surveillance System (BRFSS), a monthly state-based trend survey conducted over the telephone. Participants were adult respondents in the 50 US states and District of Columbia who were surveyed March to December 2020 compared with the same months in 2017 to 2019.

**Exposures:**

Monthly state COVID-19 death rates.

**Main Outcomes and Measures:**

Estimated 30-day prevalence of clinically significant anxiety and depression based on responses to a single BRFSS item calibrated to a score of 6 or greater on the 4-item Patient Health Questionnaire (area under the receiver operating characteristic curve, 0.84). All percentages are weighted based on BRFSS calibration weights.

**Results:**

Overall, there were 1 429 354 respondents, with 1 093 663 in 2017 to 2019 (600 416 [51.1%] women; 87 153 [11.8%] non-Hispanic Black; 826 334 [61.5%] non-Hispanic White; 411 254 [27.8%] with college education; and 543 619 [56.8] employed) and 335 691 in 2020 (182 351 [51.3%] women; 25 517 [11.7%] non-Hispanic Black; 250 333 [60.5%] non-Hispanic White; 130 642 [29.3%] with college education; and 168 921 [54.9%] employed). Median within-state response rates were 45.9% to 49.4% in 2017 to 2019 and 47.9% in 2020. Estimated 30-day prevalence of clinically significant anxiety and depression was 0.4 (95% CI, 0.0 to 0.7) percentage points higher in March to December 2020 (12.4%) than March to December 2017 to 2019 (12.1%). This estimated increase was limited, however, to students (2.4 [95% CI, 0.8 to 3.9] percentage points) and the employed (0.9 [95% CI, 0.5 to 1.4] percentage points). Estimated prevalence decreased among the short-term unemployed (−1.8 [95% CI, −3.1 to −0.5] percentage points) and those unable to work (−4.2 [95% CI, −5.3 to −3.2] percentage points), but did not change significantly among the long-term unemployed (−2.1 [95% CI, −4.5 to 0.5] percentage points), homemakers (0.8 [95% CI, −0.3 to 1.9] percentage points), or the retired (0.1 [95% CI, −0.6 to 0.8] percentage points). The increase in anxiety and depression prevalence among employed people was positively associated with the state-month COVID-19 death rate (1.8 [95% CI, 1.2 to 2.5] percentage points when high and 0.0 [95% CI, −0.7 to 0.6] percentage points when low) and was elevated among women compared with men (2.0 [95% CI, 1.4 to 2.5] percentage points vs 0.2 [95% CI, −0.1 to 0.6] percentage points), Non-Hispanic White individuals compared with Hispanic and non-Hispanic Black individuals (1.3 [95% CI, 0.6 to 1.9] percentage points vs 1.1 [95% CI, −0.2 to 2.5] percentage points and 0.7 [95% CI, −0.1 to 1.5] percentage points), and those with college educations compared with less than high school educations (2.5 [95% CI, 1.9 to 3.1] percentage points vs −0.6 [95% CI, −2.7 to 1.4] percentage points).

**Conclusions and Relevance:**

In this survey study, clinically significant US adult anxiety and depression increased less during 2020 than suggested by online surveys. However, this modest aggregate increase could mask more substantial increases in key population segments (eg, first responders) and might have become larger in 2021 and 2022.

## Key Points

**Question:**

How much did clinically significant anxiety and depression increase among US adults during the first year of the COVID-19 pandemic?

**Findings:**

In this survey study of more than 1.4 million respondents in the US Behavioral Risk Factor Surveillance System survey, responses to a screening question calibrated to a 4-item Patient Health Questionnaire score of 6 or greater suggested that aggregate prevalence of clinically significant anxiety and depression increased only modestly overall among US adults in 2020 compared with 2017 to 2019.

**Meaning:**

This modest estimated aggregate increase could mask more substantial increases in key population segments (eg, first responders) and might have become larger in 2021 and 2022.

## Teaser

This survey study examines trend data in a calibrated screening for clinically significant anxiety and depression among adults during the first year of the pandemic in the only US government benchmark probability trend survey not disrupted by the COVID-19 pandemic, the Behavioral Risk Factor Surveillance System (BRFSS).

## Introduction

Concerns about adverse mental health effects of COVID-19 have been raised since the beginning of the pandemic^1,2^ based on evidence for such outcomes in past infectious disease outbreaks^3,4,5^ and natural disasters.^6^ Many empirical papers subsequently investigated the association of the pandemic with mental health,^7^ and most concluded that the pandemic caused dramatic increases in anxiety and depression. However, such studies mostly compared online pandemic-era surveys with low or unreported response rates against prepandemic government benchmark probability surveys. These comparisons could be biased. For example, Twenge and Joiner^8^ surveyed 19 330 adults in April 2020 in an online nonprobability consumer panel^9^ using the same self-reported screening for serious mental illness (SMI)^10^ as the 2018 National Health Interview Survey (NHIS)^11^ and concluded that SMI prevalence increased 8-fold since the 2018 NHIS. Although the consumer survey was weighted to match the population on basic geographic and sociodemographic characteristics, it might have been quite different on psychological characteristics.^12^ The notion that these design differences could influence prevalence estimates is suggested by a survey carried out prior to the pandemic (early 2019) in the RAND American Life Panel (ALP),^13^ another online consumer panel with an unreported response rate. SMI had an estimated prevalence nearly 3 times as high as the NHIS estimate.^14^

Numerous other reports based on similar comparisons concluded that the pandemic caused massive increases in anxiety and depression.^8,15,16,17,18^ Included here were studies using the US Centers for Disease Control and Prevention (CDC) Household Pulse Survey (HPS),^19^ a US government trend survey started in April 2020 to track effects of COVID-19 on US residents. The HPS contains the 4-item Patient Health Questionnaire (PHQ-4) anxiety and depression screening scale,^20^ a scale also used in the NHIS and in a subsample of another important national government benchmark survey, the CDC Behavioral Risk Factor Surveillance System (BRFSS).^21^ Comparisons of HPS with 2018 NHIS and BRFSS found 3.0 to 5.0 elevated relative risk (RR) of PHQ-4 scores greater than 6.^15,22^

Importantly, the HPS, unlike the NHIS or BRFSS, is a Census Bureau Experimental Data Product^23^ used for rapid response to time-sensitive questions before more definitive results are available. It uses an online survey with only a 2% to 10% response rate.^24^ Census Bureau documentation states clearly that it is hazardous to compare HPS results with those in earlier benchmark government surveys because of the low HPS response rate.^25^ The potential biases of relying on a large sample (eg, 1 660 957 respondents in the 2020 HPS) with low a response rate was amply illustrated in a recent *Nature* report documenting that the HPS substantially overestimated early COVID-19 vaccine uptake compared with criterion standard data subsequently assembled by the CDC.^26^

More accurate before-during comparisons require either cohort studies (ie, the same individuals assessed over time) with high follow-up response rates or trend studies (ie, the same sampling and field procedures applied to different individuals) with high and comparable before-during response rates. Illustrating what a difference such designs can make, a worldwide review of data on prevalence of common mental disorders across 33 cohort studies with high follow-up response rates carried out both shortly before and in the first year of the pandemic^27^ concluded that general population mental health was not worse in 2020 than in pre–COVID-19 surveys.^28^ However, only 3 of the 33 studies were from the United States: a convenience sample of gender minority adults and 2 small samples of university students, all finding small increases in screening scale scores of anxiety or depression.^29^ Two reports from the RAND ALP panel also assessed respondents the year before and then again early in the pandemic and found similarly small increases in depression^30^ and SMI.^14^ These results are consistent with small increased prevalence of clinically significant anxiety and depression in the US adult population during 2020.^29^ A recent *Lancet* report based on a cross-national meta-analysis arrived at a similar conclusion^31^ but again with only 3 before-during US comparison studies with weak designs.^30,32,33^ A more recent meta-analysis using a larger number of mostly convenience samples came to a similar conclusion.^34^

Aggregation of results across such samples provides only a weak basis for drawing conclusions about the true pandemic effect on population mental health. As noted previously, US government benchmark trend surveys produce much more accurate information. However, most of these are face-to-face surveys that were disrupted by the pandemic in 2020. The BRFSS, in comparison, is a telephone survey that continued throughout 2020.^21^ The 2020 BRFSS microdata have recently been released, making it possible to estimate pandemic-associated changes in prevalence of clinically significant anxiety and depression in the first 10 months of the pandemic vs earlier years. We present such an analysis in this article, examining trends in the total adult population and subgroups.

## Methods

The study followed the American Association for Public Opinion Research (AAPOR) reporting guideline for survey studies.^35^ Because all data were obtained from public sources, this study does not constitute human participant research and does not require institutional review board review or exemption according to the US Department of Health and Human Services (45 CFR §46).

### Sample

The BRFSS is a collaboration between the CDC and US states to implement monthly telephone surveys of noninstitutionalized adults on prevalence-correlates of chronic conditions, risk behaviors, and outcomes of state policies^21^ based on a dual-frame landline and cell phone design.^36^ Complex calibration weights are used to adjust for discrepancies between the sample and population on a wide range of sociodemographic and geographic characteristics^36^ The median state-level response rate in 2020 was 47.9%,^37^ using the AAPOR R4 definition,^38^ very similar to 2017 to 2019 (45.9%-49.4%),^39,40,41^ the prepandemic years used for comparison. We focus on 335 691 respondents in the 50 states and District of Columbia surveyed in March to December 2020 compared with 1 093 663 respondents surveyed in the same months in 2017 to 2019.

### Measures

#### Estimated Pandemic-Associated Prevalence of Clinically Significant Anxiety and Depression

Although the core BRFSS does not include anxiety or depression screening scales, a 2018 BRFSS module administered to 8724 participants included the PHQ-4, the same anxiety and depression screening scale used in the HPS and some other major online COVID-19 population surveys.^15,42^ Clinically significant anxiety and depression are conventionally estimated as a PHQ-4 score of 6 or greater.^43^ In a preliminary analysis, we found that responses to a core BRFSS question (“Now thinking about your mental health, which includes stress, depression, and problems with emotions, for how many days during the past 30 days was your mental health not good?”) had good concordance with this dichotomous version of the PHQ-4 (area under the receiver operating characteristic curve, 0.84) in the BRFSS module sample. We consequently dichotomized responses to the core BRFSS question at 15 to 30 days vs 0 to 14 days, the threshold that best equalized false positives and false negatives for a PHQ-4 score of 6 or greater (7.2% vs 6.2%) and used this dichotomy as the outcome in our analysis.

#### Sociodemographic Characteristics

Sociodemographic characteristics included gender (female, male, or missing), race and ethnicity (Hispanic, non-Hispanic Black, non-Hispanic White, other, or missing), education (less than high school or missing, high school graduate, some college, or college graduate), and employment status. Separate questions were asked about Hispanic ethnicity (“Are you Hispanic, Latino/a, or Spanish Origin?”) and race (“Which one or more of the following would you say is your race?”). The response options for race were American Indian or Alaskan Native, Asian, Black or African American, Pacific Islander, and White*.* Respondents could endorse any or all these responses. Other volunteered responses were field coded as “Other,” “Don’t know/Not sure,” and “Refused.” Responses were coded hierarchically for the current analysis into Hispanic, Non-Hispanic Black, those reporting only White, and all others. Importantly, given the dramatic increase in unemployment early in the pandemic,^44^ which might have induced a healthy worker effect, the BRFSS assessed long-term (≥12 months) unemployment (ie, unemployment occurring before the pandemic for 2020 respondents) separately from shorter-term unemployment. These sociodemographic characteristics were included in analyses based on a suggestion in the literature that they might be associated with anxiety and depression via increased risk of virus exposure.^45^

#### Time and Space Pandemic-Associated Variables

The public use BRFSS data set includes information about state of residence and month of survey, allowing respondents to be classified on state-month COVID-19 death rate^46^ and unemployment rate^47^ compared with 2017 to 2019. These scores were assigned to all BRFSS respondents for the month before the survey.

### Statistical Analysis

We began by inspecting a cross-tabulation of bimonthly March to December anxiety and depression prevalence estimates in BRFSS 2020 vs 2017 to 2019 overall and in employment status subgroups. A modified Poisson regression model with robust variance estimation for a dichotomous outcome was then used to estimate pandemic-associated prevalence of clinically significant anxiety and depression within employment subsamples using dummy-coded variables for 2020 vs 2017 to 2019, controlling state, month, and sociodemographic characteristics. This model was then expanded to include interactions of the 2020 vs 2017 to 2019 indicator variable with sociodemographic characteristics. Temporal changes in prevalence were modeled as adjusted risk differences (ARDs)^48^ using postregression transformations that compared differences in mean estimated probabilities based on nonlinear models.^49^ Adjustments for spatial autocorrelation (ie, clustering by state) and calibration weights on SEs were made using the Taylor series linearization method.^50^ All analyses were carried out using Stata software version 16.1 (StataCorp). Statistical significance was set at *P* < .05, and all tests were 2-tailed.

## Results

### Sample Composition

Overall, there were 1 429 354 respondents, with 1 093 663 in 2017 to 2019 (600 416 [51.1%] women; 87 153 [11.8%] non-Hispanic Black; 826 334 [61.5%] non-Hispanic White; 411 254 [27.8%] with college education; and 543 619 [56.8] employed) and 335 691 in 2020 (182 351 [51.3%] women; 25 517 [11.7%] non-Hispanic Black; 250 333 [60.5%] non-Hispanic White; 130 642 [29.3%] with college education; and 168 921 [54.9%] employed) (Table 1). Compositional changes were very small, other than for a decrease in the proportion employed (1.9 percentage points; 54.9% vs 56.8%) and a significant increase in the proportion short-term unemployed (3.1 percentage points; 5.8% vs 2.7%) in 2020.

**Table 1.** Demographic Characteristics of the Sample

Characteristics	Respondents, No. (%)^a^	
	March-December 2017-2019 (n = 1 093 663)	March-December 2020 (n = 335 691)
Gender		
Female	600 416 (51.1)	182 351 (51.3)
Male	493 247 (48.9)	153 340 (48.7)
Race and ethnicity^b^		
Hispanic	83 233 (16.4)	27 162 (16.8)
Non-Hispanic		
Black	87 153 (11.8)	25 517 (11.7)
White	826 334 (61.5)	250 333 (60.5)
Other	96 943 (10.4)	32 679 (11.0)
Education		
College	411 254 (27.8)	130 642 (29.3)
High school graduate	598 712 (58.6)	182 054 (58.0)
Less than high school	83 697 (13.6)	22 995 (12.6)
Employment		
Employed	543 619 (56.8)	168 921 (54.9)
Unemployed		
Short-term	21 399 (2.7)	14 641 (5.8)
Long-term	21 915 (2.5)	5906 (2.3)
Unable to work	80 207 (6.9)	20 869 (6.2)
Student	28 451 (5.4)	8921 (5.1)
Homemaker	53 802 (5.9)	13 675 (4.8)
Retired	331 471 (18.3)	96 680 (18.8)
Missing	12 799 (1.5)	6078 (2.1)
Total, March-December	1 093 663 (100.0)	335 691 (100.0)

^a^ Sample sizes are unweighted, and percentage estimates are based on Behavioral Risk Factor Surveillance System calibration weights designed to adjust for discrepancies between the sample and population within and across states on a range of sociodemographic and geographic variables.^36^

^b^ Details of the categorization of race and ethnicity appear in the Methods section.

### Estimated Prevalence of Clinically Significant Anxiety and Depression in 2017 to 2019 and 2020

Estimated prevalence of clinically significant anxiety and depression was 0.4 (95% CI, 0.0 to 0.7) percentage points higher in March to December 2020 than March to December 2017 to 2019 (12.4% vs 12.1%). However, this difference varied significantly by month (Table 2), with estimated prevalence significantly lower in March to April 2020 than March to April 2017 to 2019 (−0.8 [95% CI, −1.6 to −0.0] percentage points; 10.9% vs 11.7%), not meaningfully different in May to June (0.2 [95% CI, −0.5 to 0.9] percentage points; 12.5% vs 12.3%), and significantly higher by increasing proportions than in 2017 to 2019 between July and August 2020 (0.7 [95% CI, 0.1 to 1.3] percentage points; 12.6% vs 11.9%) and November-December 2020 (1.0 [95% CI, 0.4 to 1.5] percentage points; 13.4% vs 12.4%).

**Table 2.** Thirty-Day Estimated Prevalence of Clinically Significant Anxiety and Depression During the First 10 Months of the COVID-19 Pandemic Compared With the Same Months in 2017-2019

Month	No. with clinically significant anxiety and depression/total No. (%)^a^		Difference, % (95% CI)^b^
	2017-2019	2020	
March-April	22 557/209 386 (11.7)	7598/78 273 (10.9)	−0.8 (−1.6 to −0.0)^c^
May-June	25 029/225 532 (12.3)	7404/65 173 (12.5)	0.2 (−0.5 to 0.9)
July-August	24 418/227 688 (11.9)	7035/61 649 (12.6)	0.7 (0.1 to 1.3)^c^
September-October	24 166/218 977 (12.2)	7311/59 252 (13.1)	0.9 (−0.0 to 1.8)
November-December	23 496/212 080 (12.4)	9181/71 344 (13.4)	1.0 (0.4 to 1.5)^c^
Total, March-December all years	119 666/1 093 663 (12.1)	38 529/335 691 (12.4)	0.4 (0.0 to 0.7)^c^

^a^ Sample sizes and count of respondents with clinically significant anxiety and depression are unweighted, and percentage estimates are based on Behavioral Risk Factor Surveillance System calibration weights designed to adjust for discrepancies between the sample and population within and across states on a range of socio-demographic and geographic variables.^36^

^b^ The 95% CIs are based on weighted data and take into consideration the spatial autocorrelation of the Behavioral Risk Factor Surveillance System sample design with cluster by state.

^c^ Significantly different from 2017 to 2019 at the .05 level based on a design-adjusted 2-sided test.

### Variation by Employment Status

Inspection of sample composition by employment status showed, consistent with official government statistics,^51^ that short-term unemployment was twice as common in 2020 as in 2017-2019 (3.1 [95% CI, 2.9 to 3.2] percentage points; 5.8% vs 2.7%) whereas employment was significantly less common (−1.9 [95% CI, −2.4 to 1.3] percentage points; 54.9% vs 56.8%) (Table 3). Differences in other employment statuses were much smaller. Estimated prevalence of clinically significant anxiety and depression increased significantly only among the employed (0.9 [95% CI, 0.5 to 1.4] percentage points; 10.8% vs 9.8%) and students (2.4 [95% CI, 0.8 to 3.9] percentage points; 17.3% vs 14.9%). In comparison, the 2020 vs 2017 to 2019 estimated change in anxiety and depression prevalence was negative among the short-term unemployed (−1.8 [95% CI, −3.1 to −0.5] percentage points; 19.2% vs 21.0%) and those unable to work (−4.2 [95% CI, −5.3 to −3.2] percentage points; 31.0% vs 35.2%) and nonsignificant among the long-term unemployed (−2.1 percentage points; 20.7% vs 22.8%), homemakers (0.8 [95% CI, −0.3 to 1.9] percentage points; 11.7% vs 10.9%), and the retired (0.1 [95% CI, −0.6 to 0.8] percentage points; 7.4% vs 7.3%).

**Table 3.** Distribution of Employment Status and 30-Day Estimated Prevalence of Clinically Significant Anxiety and Depression During the First 10 Months of the COVID-19 Pandemic Compared With the Same Months in 2017-2019 Separately by Employment Status

Employment status	Distribution			30-d estimated prevalence of anxiety and depression^a^		
	No. (%)^b^		Difference (95% CI)^c^	No. (%)^b^		Difference, % (95% CI)^c^
	2017-2019	2020		2017-2019	2020	
Employed	543 619 (56.8)	168 921 (54.9)	−1.9 (−2.4 to −1.3)^d^	47 644 (9.8)	16 989 (10.8)	0.9 (0.5 to 1.4)^d^
Unemployed						
Short-term	21 399 (2.7)	14 641 (5.8)	3.1 (2.9 to 3.2)^d^	4600 (21.0)	2903 (19.2)	−1.8 (−3.1 to −0.5)^d^
Long-term	21 915 (2.5)	5906 (2.3)	−0.2 (−0.3 to −0.0)^d^	5126 (22.8)	1309 (20.7)	−2.1 (−4.7 to 0.5)
Unable to work	80 207 (6.9)	20 869 (6.2)	−0.8 (−1.1 to −0.4)^d^	28 417 (35.2)	6679 (31.0)	−4.2 (−5.3 to −3.2)^d^
Student	28 451 (5.4)	8921 (5.1)	−0.3 (−0.5 to −0.2)^d^	4476 (14.9)	1565 (17.3)	2.4 (0.8 to 3.9)^d^
Homemaker	53 802 (5.9)	13 675 (4.8)	−1.1 (−1.4 to −0.7)^d^	5577 (10.9)	1611 (11.7)	0.8 (−0.3 to 1.9)
Retired	331 471 (18.3)	96 680 (18.8)	0.5 (0.2 to 0.9)^d^	22 514 (7.3)	6811 (7.4)	0.1 (−0.6 to 0.8)
Missing	12 799 (1.5)	6078 (2.1)	0.6 (0.5 to 0.7)^d^	1312 (11.0)	662 (10.0)	−1.0 (−2.5 to 0.5)
Total	1 093 663 (100.0)	335 691 (100.0)	NA	119 666 (12.1)	38 529 (12.4)	0.4 (0.0 to 0.7)^d^

Abbreviation: NA, not applicable.

^a^ The distribution of estimated prevalence of clinically significant anxiety and depression varied significantly with employment status both in 2017 to 2019 (F_7,51_ = 6034.5; *P* < .001) and in 2020 (F_7,51_ = 813.6; *P* < .001). In addition, the association between employment status and estimated prevalence of clinically significant anxiety and depression differed between the 2 times (F_7,51_ = 45.2; *P* < .001).

^b^ Sample sizes are unweighted, and percentage estimates are based on Behavioral Risk Factor Surveillance System calibration weights designed to adjust for discrepancies between the sample and population within and across states on a range of socio-demographic and geographic variables.^36^

^c^ The 95% CIs are based on weighted data and take into consideration the spatial autocorrelation of the Behavioral Risk Factor Surveillance System sample design with cluster by state.

^d^ Significantly different from 2017 to 2019 at the .05 level based on a design-adjusted 2-sided test.

### Time-Space Variation by Employment Status

Only 2 plausible interactions emerged from regression analyses comparing estimated pandemic-associated changes in prevalence of clinically significant anxiety and depression by employment status across sample quartiles defined by state-month COVID-19 death rates and unemployment rates (vs the same states-months in 2017-2019). Both these subgroup differences in ARD were among the employed: higher estimated increases in prevalence in state-months with elevated COVID-19 death rates and elevated unemployment rates (Table 4). However, only the first of these remained significant when both were considered together, with increases in prevalence highest in the quartile with the highest COVID-19 death rate (ARD, 1.8 [95% CI, 1.2 to 2.5] percentage points), lower in the 2 intermediate quartiles (high-average: ARD, 1.0 [95% CI, 0.7 to 1.4] percentage points; low-average: ARD, 1.1 [95% CI, 0.3 to 1.9] percentage points), and nonsignificant in the lowest quartile (ARD, −0.0 [95% CI, −0.7 to 0.6] percentage points).

**Table 4.** Pandemic-Associated Change in 30-Day Estimated Prevalence of Clinically Significant Anxiety and Depression During the First 10 Months of the COVID-19 Pandemic in Subsamples Defined by State-Month Differences in the COVID-19 Death Rate and the Unemployment Rate Compared With 2017-2019^a^

Quartile	ARD (95% CI), percentage points					No.^b^
	Employed	Unemployed	Unable to work	Students	Other	
**Overall**						
Total sample	1.0 (0.6 to 1.4)^c^	−1.7 (−3.0 to −0.3)^c^	−3.8 (−4.8 to −2.9)^c^	2.2 (1.2 to 3.2)^c^	0.2 (−0.3 to 0.7)	1 429 354
**Time-space variation in the state-month COVID-19 death rate**						
High	1.8 (1.2 to 2.5)^c^	−1.0 (−3.2 to 1.2)	−3.8 (−5.6 to −1.9)^c^	2.4 (−0.1 to 4.9)	0.1 (−0.8 to 1.0)	333 231
High-average	1.0 (0.7 to 1.4)^c^	−3.8 (−7.5 to −0.1)^c^	−2.1 (−3.9 to −0.3)^c^	0.6 (−1.7 to 3.0)	0.4 (−0.4 to 1.2)	324 347
Low-average	1.1 (0.3 to 1.9)^c^	0.1 (−2.6 to 2.7)	−4.3 (−7.1 to −1.4)^c^	4.0 (1.4 to 6.7)^c^	0.4 (−0.6 to 1.3)	346 247
Low	−0.0 (−0.7 to 0.6)	−2.3 (−5.0 to 0.4)	−4.2 (−6.1 to −2.3)^c^	1.4 (−1.1 to 3.8)	0.0 (−0.6 to 0.6)	425 529
F_3,51_	11.6^d^	2.5	1.1	1.3	0.3	NA
**Time-space variation in the state-month 2020 unemployment rate compared with the same month in 2017-2019**						
High	1.3 (0.9 to 1.7)^c^	−2.6 (−4.5 to −0.7)^c^	−3.8 (−6.4 to −1.2)^c^	0.8 (−1.5 to 3.0)	0.3 (−0.8 to 1.4)	254 287
High-average	1.3 (0.3 to 2.3)^c^	−0.6 (−3.3 to 2.1)	−3.6 (−5.6 to −1.6)^c^	4.5 (2.7 to 6.4)^c^	0.5 (−0.5 to 1.6)	332 021
Low-average	1.2 (0.8 to 1.7)^c^	−1.9 (−3.8 to −0.1)^c^	−3.2 (−5.2 to −1.3)^c^	1.5 (−0.7 to 3.7)	0.0 (−0.7 to 0.7)	376 099
Low	0.2 (−0.5 to 0.8)	−1.9 (−4.9 to 1.2)	−3.7 (−5.7 to −1.6)^c^	1.8 (−1.0 to 4.6)	0.2 (−0.4 to 0.7)	466 947
F_3,51_	3.6^d^	0.8	0.1	2.9^d^	0.2	NA

Abbreviations: ARD, adjusted risk difference; NA, not applicable.

^a^ Results in the table are based on a model with 51 dummy indicator variables for states, 9 for months, 3 for race and ethnicity, 3 for education, 1 for gender, 2 for age, 3 for marital status, 3 for number of children in household, 4 for quartile of state-month COVID-19 death rate or unemployment rate, and 4 subgroup-coded interactions for the 2020 indicator variable within the COVID-19 death rate or unemployment rate quartile. The model was estimated in weighted data using the Behavioral Risk Factor Surveillance System calibration weights.^36^ The total sample row corresponds to a single model estimated on the entire sample. The subsequent results correspond to a single model estimated in subsamples defined by employment status. The values shown in the table are the ARDs^48^ from postregression transformations of the coefficients for the 4 interactions of quartile of state-month COVID-19 death rate or unemployment rate with the 2020 indicator variable.

^b^ Sample sizes are unweighted.

^c^ Significantly different from 2017 to 2019 at the .05 level based on a design-adjusted 2-sided test.

^d^ Significant difference in estimated pandemic-associated changes in prevalence of clinically significant anxiety and depression across quartiles of state-month COVID-19 death rate or unemployment rate based on .05 level design-adjusted 2-sided tests.

We tested whether the association of employment and COVID-19 death rate varied by sociodemographic characteristics and found that there were differences only for gender (Table 5). The aggregate ARD differed significantly between women and men, was significant only among women (women: ARD, 2.0 [95% CI, 1.4 to 2.5] percentage points; men: 0.2 [95% CI, −0.1 to 0.6] percentage points) and varied significantly with the state-month COVID-19 death rate among women (high: ARD, 3.3 [95% CI, 2.3 to 4.2 percentage points]; low: ARD, 0.1 [−1.0 to 1.2] percentage points). The aggregate ARD also differed significantly across subgroups defined by race and ethnicity but was significant only among Non-Hispanic White respondents (ARD, 1.3% [95% CI, 0.6 to 1.9] percentage points; Hispanic respondents: 1.1 [95% CI, −0.2 to 2.5] percentage points; non-Hispanic Black respondents: 0.7 [95% CI, −0.1 to 1.5] percentage points) and varied significantly with the state-month COVID-19 death rate in that subsample (high: ARD, 2.6 [95% CI, 1.8 to 3.4] percentage points; low: ARD, 0.5 [95% CI, −0.4 to 1.5] percentage points). Finally, the aggregate ARD differed significantly across subgroups defined by level of education but was significant only among those with a college degree (college degree: ARD, 2.5 [95% CI, 1.9 to 3.1] percentage points; <high school: ARD, −0.6 [95% CI, −2.7 to 1.4] percentage points) and varied significantly with the state-month COVID-19 death rate in that subsample (high: ARD, 3.1 [2.3 to 4.0] percentage points; low: 1.7 [0.3 to 3.1] percentage points).

**Table 5.** Variation in Pandemic-Associated Change in Estimated 30-Day Prevalence of Clinically Significant Anxiety and Depression During the First 10 Months of the COVID-19 Pandemic Among the Employed in Subgroups Defined by State-Month Differences and Sociodemographic Variables^a^

Variable	State-month COVID-19 death rate, ARD (95% CI), percentage points^b^				F_2,51_
	Total	High	Intermediate	Low	
Gender					
Female	2.0 (1.4 to 2.5)^c^	3.3 (2.3 to 4.2)^c^	2.3 (1.6 to 3.0)^c^	0.1 (−1.0 to 1.2)	16.4^d^
Male	0.2 (−0.1 to 0.6)	0.8 (−0.2 to 1.8)	0.2 (−0.2 to 0.7)	−0.2 (−0.8 to 0.4)	1.7
F_1,51_	106.3^e^	15.0^e^	62.5^e^	0.2	NA
Race and ethnicity^f^					
Hispanic	1.1 (−0.2 to 2.5)	1.2 (−0.4 to 2.9)	2.5 (0.7 to 4.4)^c^	−2.5 (−4.7 to −0.3)^c^	12.5^d^
Non-Hispanic					
Black	0.7 (−0.1 to 1.5)	0.6 (−0.8 to 1.9)	1.4 (−0.2 to 3.0)	−0.7 (−4.0 to 2.7)	0.4
White	1.3 (0.6 to 1.9)^c^	2.6 (1.8 to 3.4)^c^	1.0 (0.1 to 1.9)^c^	0.5 (−0.4 to 1.5)	11.6^d^
Other	−0.2 (−0.9 to 0.4)	−0.1 (−2.5 to 2.4)	−0.7 (−1.5 to 0.2)	0.7 (−0.6 to 1.9)	3.7^d^
F_3,51_	7.2^e^	3.4^e^	8.3^e^	2.4	NA
Education					
College-educated	2.5 (1.9 to 3.1)^c^	3.1 (2.3 to 4.0)^c^	2.6 (2.0 to 3.3)^c^	1.7 (0.3 to 3.1)^c^	3.4^d^
High school or some college	0.3 (−0.3 to 0.9)	1.6 (0.4 to 2.7)^c^	0.4 (−0.3 to 1.2)	−1.0 (−2.0 to 0.1)	7.6^d^
Less than High school	−0.6 (−2.7 to 1.4)	−0.6 (−3.5 to 2.3)	−0.3 (−3.7 to 3.2)	−1.5 (−4.2 to 1.2)	0.1
F_2,51_	24.6^e^	6.4^e^	63.2^e^	11.5^e^	NA

Abbreviations: ARD, adjusted risk difference; NA, not applicable.

^a^ Results in the total column are based on separate models for gender, race and ethnicity, and education, in each of which factors include 51 dummy indicator variables for states, 9 for months, 1 for gender, 3 for race and ethnicity, 2 for education, 3 for the state-month COVID-19 death rate quartile, and between 2 (for gender) and 4 (for race and ethnicity) subgroup-coded interactions for the 2020 indicator variable within each of the sociodemographic variable subgroups that are the focus of the model. The models were estimated in weighted data using the Behavioral Risk Factor Surveillance System calibration weights.^36^ Results in the death rate subgroup models are based on an expansion of the total sample model with between 5 (for gender) and 11 (for race and ethnicity) dummy indicator variables for the cross-classification of state-month COVID-19 death rates with the focal sociodemographic variable and between 6 (for gender) and 12 (for race and ethnicity) subgroup-coded 3-way interactions for the 2020 indicator variable within each of the subgroups. The values shown in the table are the ARDs^48^ from postregression transformations for the subgroups defined by the combination of row and column variables.

^b^ High indicates the quartile of the 2020 sample with the highest state-month COVID-19 death rate in the prior month, along with the same states in the same months in 2017 to 2019; intermediate, the 2 middle quartiles; and low, the lowest quartile.

^c^ Significantly different from 2017 to 2019 at the .05 level based on a design-adjusted 2-sided test.

^d^ Significant difference in estimated pandemic-associated change in prevalence of clinically significant anxiety and depression across subgroups defined by state-month COVID-19 death rate within a row of the table.

^e^ Significant difference in estimated pandemic-associated change in prevalence of clinically significant anxiety and depression across sociodemographic subgroups within a column of the table.

^f^ Details of the categorization of race and ethnicity appear in the Methods section.

### Sensitivity Analysis

As instability in the before-during comparisons could have occurred because of a considerably higher estimated prevalence of clinically significant anxiety and depression in 2019 than 2017-2018, analyses were replicated comparing 2020 separately with 2017 to 2018 and 2019. All key results—most notably, the estimated pandemic-associated increase in prevalence among the employed, the positive association of that increase with the state-month COVID-19 death rate, and significant estimated pandemic-associated decreases in prevalence among the unemployed and those unable to work—were replicated in the separate analyses (eTables 1-2 in the Supplement).

## Discussion

The BRFSS results raise the possibility that 2020 US adult pandemic-associated increases in clinically significant anxiety and depression were much more modest than suggested in previous studies. This is indirectly consistent with trends in contacts with the National Suicide Prevention Lifeline, which decreased in nearly as many states as they increased in quarter 2 of 2020 compared with 2019 and in ways unassociated with the state COVID-19 death rate or unemployment rate.^52^ Adult suicide deaths also decreased slightly in 2020 vs 2019.^53^

These estimated pandemic-associated increases in clinically significant anxiety and depression were smaller than expected given the substantial 2020 increases^54,55^ in stressors known to cause clinically significant psychological distress, including job loss,^56^ death of loved ones,^57^ and social isolation,^58^ particularly among disadvantaged groups (eg, those with lower educational attainment and those belonging to minoritized racial and ethnic groups), given that exposure to these factors was elevated in these segments of the population.^59,60^ Only one of these stressors, job loss, was assessed in the BRFSS. Even more surprising was that prevalence was estimated to decrease among the unemployed and those unable to work. Importantly, these decreases occurred for both short-term and long-term unemployment, arguing against a healthy worker effect.

One plausible interpretation of this specification is that anxiety and depression were associated with risk of infection when going to work, which increased as the COVID-19 death rate increased. We investigated this possibility indirectly by examining subgroup variation based on the observation that “women, people of color, and those of lower socioeconomic status” were most likely “to hold frontline positions that require in-person work and the least likely to have paid sick leave.”^45^ This suggests that (1) the elevated ARD among the employed should be higher for women than men; for Hispanic, non-Hispanic Black, and people of other races and ethnicities than non-Hispanic White respondents; and for people with lower rather than higher education and (2) the magnitude of these differences should vary positively with state-month COVID-19 death rates. However, this difference was only significant when comparing women with men.

Early psychological resilience during other infectious disease outbreaks and natural disasters has sometimes given way to subsequent increases in psychopathology as the crises become more protracted. Such a pattern is typical, for example, for suicide rates in the early and later phases of long-lasting mass traumas.^61^ The same might have occurred in the second year of the COVID-19 pandemic. Indeed, preliminary evidence consistent with this possibility has already been reported in Japan.^62^ In addition, the trend in emergency department visits for suicide attempts in the United States has been increasing among adolescents.^63^ And significant increases occurred in late 2020 both in drug overdose deaths^64^ and in rates of fatal and nonfatal domestic violence.^65^

Although it is impossible to predict future trends with accuracy, there are some promising signs in the expansion of tele–mental health care and increased use of scalable interventions to address increasing demand for treatment of emotional problems. However, there are also uncertainties about the effects of reversals in federal safety net policies to reduce the financial impact of the pandemic on the most vulnerable segments of the population^66^ and lingering uncertainties about the duration of the pandemic and the possible long-term neuropsychiatric effects of infection.^67^ As noted previously, prior research suggests that prolongation of disasters and proliferation of secondary stressors can have severe negative effects on mental health.^68,69^

Public policy decisions require better and more timely data than those produced by the online surveys that have been the mainstay of research on pandemic-associated changes in anxiety and depression or by using responses to the single question we were forced to rely on in the BRFSS. Methods exist to generate considerably better estimates but require a commitment to developing durable calibration rules prior to the next mass trauma to link the results of high-quality probability surveys with the results of more practical online rapid response surveys.^70,71,72^ Importantly, these calibration rules need to be geared to the focus of the surveys. This means that calibration rules appropriate for online surveys to track, eg, likely voting preferences, could be quite different from those appropriate for online surveys to track changes in clinically significant psychopathology. One would hope that the appropriate federal agencies take steps to produce such durable calibration rules before the next time they are needed.

### Limitations

This study has limitations. Our results are limited by reliance on responses to a single BRFSS question to estimate clinically significant anxiety and depression, although responses to that question had good concordance with a standard anxiety and depression screening scale. Another limitation is that the BRFSS response rate (53.3%), although considerably higher than the HPS (2%-10%), still would allow substantial bias. However, given that face-to-face benchmark government surveys were compromised during 2020, the BRFSS data are likely the best we will get for 2020. That said, it is noteworthy that the broad comparisons examined here do not rule out substantial increases in clinically significant anxiety and depression in smaller, but important, population segments, such as health workers,^73^ or population segments such as the homeless and those living in institutional settings not covered by the BRFSS. Also noteworthy is that our focus on clinically significant anxiety and depression (ie, calibration to PHQ-4 score of ≥6) leaves open the possibility of more substantial increases in less severe anxiety and depression.^29^ A final noteworthy limitation is that even though the BRFSS did not assess children, the high estimated pandemic-associated increase in prevalence of clinically significant anxiety and depression among students is consistent with administrative trend data^63^ suggesting that the effects of the pandemic on population mental health in 2020 were much more pronounced among youth than adults.

## Conclusions

In this survey study, clinically significant US adult anxiety and depression increased less during 2020 than suggested by online surveys. However, this modest aggregate increase could mask more substantial increases in key population segments and might have become larger in 2021 and 2022.

## Notes

Supplement. **eTable 1.** Estimated Pandemic-Associated 30-Day Prevalence of Clinically Significant Anxiety and Depression During the First 10 Months of the COVID-19 Pandemic in Subsamples Defined by State-Month Differences in the COVID-19 Death Rate and the Unemployment Rate Compared With 2017-2018 **eTable 2.** Estimated Pandemic-Associated 30-Day Prevalence of Clinically Significant Anxiety and Depression During the First 10 Months of the COVID-19 Pandemic in Subsamples Defined by State-Month Differences in the COVID-19 Death Rate and the Unemployment Rate Compared With 2019
