# Supplementary material for: Estimated Prevalence of and Factors Associated With Clinically Significant Anxiety and Depression Among US Adults During the First Year of the COVID-19 Pandemic
Source: JAMA Netw Open. 2022 Jun 15;5(6):e2217223. doi: 10.1001/jamanetworkopen.2022.17223 (PMC9201669; doi:10.1001/jamanetworkopen.2022.17223)
Supplement: Supplement. — eTable 1. Estimated Pandemic-Associated 30-Day Prevalence of Clinically Significant Anxiety and Depression During the First 10 Months of the COVID-19 Pandemic in Subsamples Defined by State-Month Differences in the COVID-19 Death Rate and the Unemployment Rate Compared With 2017-2018 eTable 2. Estimated Pandemic-Associated 30-Day Prevalence of Clinically Significant Anxiety and Depression During the First 10 Months of the COVID-19 Pandemic in Subsamples Defined by State-Month Differences in the COVID-19 Death Rate and the Unemployment Rate Compared With 2019 [file jamanetwopen-e2217223-s001.pdf]

Supplemental Online Content

Kessler RC, Ruhm CJ, Puac-Polanco V, et al. Estimated prevalence of and factors associated with clinically significant anxiety and depression among US adults during the first year of the COVID-19 pandemic. *JAMA Netw Open*. 2022;5(6):e2217223. doi:10.1001/jamanetworkopen.2022.17223

eTable 1. Estimated Pandemic-Associated 30-Day Prevalence of Clinically Significant Anxiety and Depression During the First 10 Months of the COVID-19 Pandemic in Subsamples Defined by State-Month Differences in the COVID-19 Death Rate and the Unemployment Rate Compared With 2017-2018

eTable 2. Estimated Pandemic-Associated 30-Day Prevalence of Clinically Significant Anxiety and Depression During the First 10 Months of the COVID-19 Pandemic in Subsamples Defined by State-Month Differences in the COVID-19 Death Rate and the Unemployment Rate Compared With 2019

This supplemental material has been provided by the authors to give readers additional information about their work.

© 2022 Kessler RC et al. *JAMA Network Open.*

eTable 1. Estimated Pandemic-Associated 30-Day Prevalence of Clinically Significant Anxiety and Depression During the First 10 Months of the COVID-19 Pandemic in Subsamples Defined by State-Month Differences in the COVID-19 Death Rate and the Unemployment Rate Compared With 2017-2018[a]

	Employed		Unemployed		Unable to work		Students		Others		
	ARD	(95% CI)	ARD	(95% CI)	ARD	(95% CI)	ARD	(95% CI)	ARD	(95% CI)	N[b]
I. Total sample	1.4[c]	(1.0, 1.8)	-1.0	(-2.4, 0.5)	-3.6[c]	(-4.7, -2.5)	3.3[c]	(2.4, 4.2)	0.3	(-0.2, 0.7)	1,076,387
II. Time-space variation in the state-month COVID-19 death rate											
High	2.2[c]	(1.5, 3.0)	-0.5	(-3.1, 2.2)	-3.2[c]	(-5.0, -1.4)	3.5[c]	(1.0, 6.1)	0.2	(-0.6, 1.0)	255,769
High-average	1.5[c]	(1.1, 1.9)	-3.5	(-7.9, 0.9)	-1.5	(-3.9, 0.8)	2.3	(-0.3, 4.8)	0.6	(-0.2, 1.4)	239,974
Low-average	1.5[c]	(0.6, 2.3)	0.6	(-1.7, 2.9)	-3.6[c]	(-6.7, -0.5)	4.5[c]	(2.0, 7.1)	0.3	(-0.5, 1.1)	259,590
Low	0.3	(-0.3, 0.9)	-0.6	(-3.0, 1.8)	-4.4[c]	(-6.8, -2.1)	2.1	(-0.7, 5.0)	0.1	(-0.5, 0.7)	321,054
$F_{3,51}$	13.7[d]		1.9		1.2		0.5		0.5		
III. Time-space variation in the state-month 2020 unemployment rate compared to the same month in 2017-2019											
High	1.7[c]	(1.3, 2.1)	-1.7	(-3.8, 0.3)	-3.0	(-6.4, 0.5)	2.1	(-0.4, 4.7)	0.5	(-0.6, 1.6)	190,190
High-average	1.6[c]	(0.6, 2.7)	0.1	(-3.0, 3.3)	-2.5[c]	(-5.0, -0.1)	4.8[c]	(3.3, 6.3)	0.6	(-0.2, 1.4)	250,293
Low-average	1.6[c]	(1.2, 2.1)	-1.9	(-3.9, 0.1)	-3.4[c]	(-5.6, -1.2)	2.9[c]	(0.4, 5.4)	-0.1[c]	(-0.7, 0.6)	281,710
Low	0.5	(-0.1, 1.2)	-0.4	(-3.4, 2.5)	-3.8[c]	(-6.2, -1.5)	2.6	(-0.2, 5.5)	0.3	(-0.2, 0.8)	354,194
$F_{3,51}$	3.8[d]		1.1		0.2		2.2		0.4		

Abbreviations: ARD, adjusted risk difference; CI, confidence interval.
[a]Results are based on models similar to those in Table 4 other than that the comparisons are between 2017-2018 and 2020 rather than between 2017-2019 and 2020.
[b]Sample sizes are unweighted
[c]Significantly different from 2017-2018 at the .05 level based on design-adjusted two-sided tests.
[d]Significant difference in estimated pandemic-related changes in prevalence of clinically significant anxiety-depression across quartiles of state-month COVID-19 death rate or unemployment rate based on .05 level design-adjusted two-sided tests.

© 2022 Kessler RC et al. *JAMA Network Open.*

eTable 2. Estimated Pandemic-Associated 30-Day Prevalence of Clinically Significant Anxiety and Depression During the First 10 Months of the COVID-19 Pandemic in Subsamples Defined by State-Month Differences in the COVID-19 Death Rate and the Unemployment Rate Compared With 2019[a]

	Employed		Unemployed		Unable to work		Students		Others		
	ARD	(95% CI)	ARD	(95 % CI)	ARD	(95% CI)	ARD	(95% CI)	ARD	(95% CI)	N[b]
I. Total sample	0.2	(-0.2, 0.6)	-3.3[c]	(-5.3, -1.2)	-4.3[c]	(-5.7, -3.0)	-0.1	(-1.7, 1.5)	0.1	(-0.6, 0.8)	688,658
II. Time-space variation in the state-month COVID-19 death rate											
High	1.0[c]	(0.3, 1.8)	-1.9	(-4.6, 0.8)	-4.4[c]	(-7.3, -1.6)	-0.4	(-3.0, 2.3)	0.0	(-1.3, 1.2)	156,290
High-average	0.1	(-0.4, 0.7)	-4.3[c]	(-7.8, -0.7)	-3.3[c]	(-5.0, -1.6)	-2.6	(-5.5, 0.2)	0.1	(-0.9, 1.2)	159,737
Low-average	0.3	(-0.4, 1.1)	-1.6	(-6.1, 2.9)	-5.7[c]	(-8.8, -2.7)	2.7	(-1.1, 6.5)	0.6	(-0.6, 1.8)	162,154
Low	-0.6	(-1.3, 0.0)	-6.2[c]	(-10.4, -2.1)	-3.6[c]	(-5.9, -1.3)	-0.4	(-3.5, 2.7)	0.0	(-0.8, 0.7)	210,477
$F_{3,51}$	6.4[d]		2.3		0.9		2.4		0.6		
III. Time-space variation in the state-month 2020 unemployment rate compared to the same month in 2017-2019											
High	0.6[c]	(0.1, 1.2)	-4.5[c]	(-7.5, -1.6)	-5.2[c]	(-7.9, -2.6)	-2.1	(-4.6, 0.4)	0.2	(-1.0, 1.4)	120,225
High-average	0.4	(-0.4, 1.2)	-2.5	(-5.4, 0.3)	-5.9[c]	(-8.3, -3.6)	3.6[c]	(0.7, 6.5)	0.5	(-1.1, 2.0)	150,850
Low-average	0.5	(-0.3, 1.2)	-1.6	(-4.5, 1.2)	-2.9[c]	(-5.0, -0.8)	-1.9	(-4.2, 0.5)	0.3	(-0.8, 1.4)	181,987
Low	-0.6	(-1.3, 0.0)	-4.9[c]	(-9.2, -0.5)	-3.1[c]	(-5.6, -0.7)	-0.2	(-3.9, 3.6)	-0.1	(-0.9, 0.8)	235,596
$F_{3,51}$	3.5[d]		0.9		2.0		4.4[d]		0.2		

Abbreviations: ARD, adjusted risk difference; CI, confidence interval.

[a]Results are based on models similar to those in Table 4 other than that the comparisons are between 2019 and 2020 rather than between 2017-2019 and 2020.

[b]Sample sizes are unweighted.

[c]Significantly different from 2019 at the .05 level based on design-adjusted two-sided tests.

[d]Significant difference in estimated pandemic-related changes in prevalence of clinically significant anxiety-depression across quartiles of state-month COVID-19 death rate or unemployment rate based on .05 level design-adjusted two-sided tests.

© 2022 Kessler RC et al. *JAMA Network Open.*
